# Unilateral traumatic brain injury of the left and right hemisphere produces the left hindlimb response in rats

**DOI:** 10.1007/s00221-021-06118-4

**Published:** 2021-05-22

**Authors:** Georgy Bakalkin, Olga Nosova, Daniil Sarkisyan, Mathias Hallberg, Mengliang Zhang, Jens Schouenborg, Niklas Marklund, Hiroyuki Watanabe

**Affiliations:** 1 Department of Pharmaceutical Biosciences, Uppsala University, Husargatan 3, Box 591, 751 24 Uppsala, Sweden; 2 Department of Molecular Medicine, University of Southern Denmark, Odense, Denmark; 3 Neuronano Research Center, Department of Experimental Medical Science, Lund University, Lund, Sweden; 4 Skåne University Hospital, Department of Clinical Sciences Lund, Neurosurgery, Lund University, Lund, Sweden

**Keywords:** Traumatic brain injury, Stroke, Ipsilateral response, Contralateral response, Postural asymmetry

## Abstract

Traumatic brain injury and stroke result in hemiplegia, hemiparesis, and asymmetry in posture. The effects are mostly contralateral; however, ipsilesional deficits may also develop. We here examined whether ablation brain injury and controlled cortical impact (CCI), a rat model of clinical focal traumatic brain injury, both centered over the left or right sensorimotor cortex, induced hindlimb postural asymmetry (HL-PA) with contralesional or ipsilesional limb flexion. The contralesional hindlimb was flexed after left or right side ablation injury. In contrast, both the left and right CCI unexpectedly produced HL-PA with flexion on left side. The flexion persisted after complete spinal cord transection suggesting that CCI triggered neuroplastic processes in lumbar neural circuits enabling asymmetric muscle contraction. Left limb flexion was exhibited under pentobarbital anesthesia. However, under ketamine anesthesia, the body of the left and right CCI rats bent laterally in the coronal plane to the ipsilesional side suggesting that the left and right injury engaged mirror-symmetrical motor pathways. Thus, the effects of the left and right CCI on HL-PA were not mirror-symmetrical in contrast to those of the ablation brain injury, and to the left and right CCI produced body bending. Ipsilateral effects of the left CCI on HL-PA may be mediated by a lateralized motor pathway that is not affected by the left ablation injury. Alternatively, the left-side-specific neurohormonal mechanism that signals from injured brain to spinal cord may be activated by both the left and right CCI but not by ablation injury.

**Supplementary Information:**

The online version contains supplementary material available at 10.1007/s00221-021-06118-4.

## Introduction

Traumatic brain injury and stroke result in asymmetric motor deficits including contralesional hemiplegia and hemiparesis and asymmetry in posture and balance (Zhang et al. 2020). Severity of impairments of contralesional limbs may be hemisphere specific and depend on side of brain lesion (Hanna-Pladdy et al. 2002; Harris and Eng 2006; Mani et al. 2013; de Paiva Silva et al. 2018). In right-handed individuals, the contralesional deficits are more severe after right hemisphere damage. Right hemisphere damage by stroke induces poorer muscular responses to stance perturbation and leads to poorer postural responses both in quiet and perturbed balance relative to left cerebral damage (Fernandes et al. 2018; Coelho et al. 2019).

Besides contralesional motor impairments, unilateral brain injuries including traumatic brain injury and stroke in humans and experimental animals result in substantial motor deficits in the ipsilateral half of the body including the ipsilesional limb (Gonzalez et al. 2004; Varghese and Winstein 2019; Maenza et al. 2020). Injury to the motor cortex in rats produced severe deficits in skilled reaching in both the contra- and ipsilateral forelimbs, suggesting that skilled movements are under bilateral control by both hemispheres (Gonzalez et al. 2004). In stroke survivors, impairments of the ipsilesional extremities depend on the side of brain lesion and vary with the severity of contralesional deficits (Varghese and Winstein 2019; Maenza et al. 2020). In patients with left hemisphere injury, ipsilesional motor deficits are proportional to the degree of impairments while this relationship is not definite in patients with injury to the right hemisphere. In other words, damage to the left hemisphere produced greater ipsilesional deficits than that to the right hemisphere.

Animal studies demonstrated that a unilateral lesion to the cerebellum and sensorimotor cortex caused asymmetric hindlimb posture (Chamberlain et al. 1963; Zhang et al. 2020), while a lateral spinal cord hemisection produced changes in spinal reflexes that paralleled asymmetry in locomotion (Hultborn and Malmsten 1983; Rossignol and Frigon 2011). Cerebellar or spinal cord injuries produced flexion or activated nociceptive withdrawal reflexes of the ipsilesional hindlimb. In contrast, the contralesional hindlimb was flexed after a localized unilateral focal lesion of the sensorimotor cortex (Lukoyanov et al. 2020; Watanabe et al. 2020) or a large hemispheric injury (Varlinskaia et al. 1984). Formation of hindlimb postural asymmetry (HL-PA) after a cortical lesion is paralleled by impairments of contralesional hindlimb function in the beam walking and ladder rung tests (Lukoyanov et al. 2020) as well in activation of hindlimb nociceptive withdrawal reflexes evoked by electrical stimulation and recorded with EMG technique on the contra- *vs*. ipsilesional side (Zhang et al. 2020). The asymmetry induced by the focal cortical injury was abolished by pancuronium, a curare-mimetic muscle relaxant suggesting a neural mechanism dependent on efferent drive. The changes in posture and reflexes induced by brain injury persisted after complete spinal cord transection suggesting that they were encoded in spinal neurocircuits (Chamberlain et al. 1963; Rossignol and Frigon 2011; Zhang et al. 2020). The HL-PA phenomenon in rats recapitulates several pathophysiological features of the human upper motor neuron syndrome including the “hemiplegic posture” and exaggerated asymmetric withdrawal reflexes and represents a reproducible and quantifiable model to unravel neurobiological mechanisms of sensorimotor and postural deficits.

A translational value of the HL-PA model is supported by the findings that the same phenomenon is induced by the controlled cortical impact (CCI), a clinically relevant focal unilateral traumatic brain injury (Watanabe et al. 2020). Interestingly, the side of the affected hindlimb in this model was not a fixed entity and was pharmacologically reversed. While the general opioid antagonist naloxone blocked HL-PA formation, administration of selective κ-opioid antagonists to the rats with the right side CCI showing the left hindlimb flexion did not affect the asymmetry magnitude but reversed the flexion side; instead of the left limb the right limb became flexed (Watanabe et al. 2020). These findings suggest that there are components of sensorimotor and postural deficits, the development of which on the contra- vs. ipsilesional side is not determined by anatomical constrains but is controlled by a hitherto unknown neurohormonal mechanism that may be unraveled in the HL-PA model.

In the course of asymmetry analysis, we unexpectedly observed that both the left and right side CCI produced HL-PA with flexion of the same, (ie; left) hindlimb that was ipsi- and contralesional to the injured hemisphere, respectively. Besides HL-PA, the CCI rats may exhibit changes in body posture including body bending to the ipsilesional side. In contrast to the left side response in the HL-PA model, changes in body posture in rats with the left and right side CCI were mirror-symmetrical. This report presents these unanticipated HL-PA and body bending findings.

## Methods

### Animals

Adult male Sprague–Dawley rats (350–400 g body weight, purchased from Taconic, Denmark) housed in standard cage with food and water ad libitum and maintained under the 12 h’ light–dark cycle at a constant environmental temperature of 21 °C (humidity: 65%) were randomly assigned to experimental groups. Animals losing more than 10% body weight following the injury were excluded from the study. All procedures were approved by the research animal ethics board of Uppsala County (permits C101/13 and C165/14) and performed according to the rules and regulations of the Swedish Board of Agriculture.

### Surgery and histology

#### Controlled cortical impact (CCI)

CCI was performed as previously described (Clausen et al. 2011; Watanabe et al. 2020). Briefly, a craniotomy, 5 × 6 mm^2^, was centered at bregma 0.5 mm and 3.5 mm lateral to the midline over the right sensorimotor cortex. CCI brain injury was induced by a CCI-device (VCU Biomedical Engineering Facility, Richmond, Virginia, USA) using a 4.0 mm diameter piston, and producing a 1.0 mm compression of the brain at a speed of 2.4 m/s and 100 ms duration. The impactor was perpendicular to the exposed cortex. For sham injury, animals underwent identical surgery and anesthesia without receiving the CCI. Animal weight was monitored and wound healing inspected daily post-surgery. No drugs were administered after brain surgery.

**Fig. 1:**
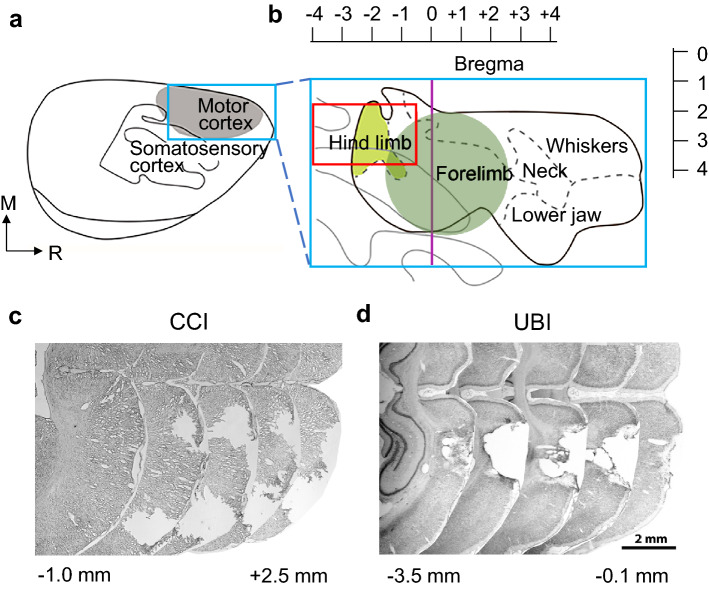
Controlled cortical impact (CCI) and unilateral ablation brain injury (UBI) of the hindlimb motor cortex. **a** Schematic representation of the sensorimotor cortex of the rat brain (modified from (Tandon et al. 2008)). **b** Expanded cortex. Green circle denotes the intended lesion area although the actual lesion area slightly varied among the rats. Red rectangle denotes the intended lesion area although the actual lesion area slightly varied among the rats. Vertical purple line indicates the bregma plane. Scales above and on the right side indicate the distance in mm relative to the bregma rostrocaudally and to the midline mediolaterally, respectively. **c** Five consecutive Nissl-stained cortical sections show the lesion site on the right cortex in two-dimension from a CCI rat. **d** Five consecutive Nissl-stained cortical sections show the lesion site on the right cortex in two-dimension from a UBI rat. Caudal is to the left and rostral is to the right for all the panels. The numbers below **c** and **d** indicate the position of the first and the last section in the figure relative to the bregma. Scale bar = 2 mm

Three days following the CCI or sham injury, rats were sacrificed with overdose of pentobarbital and the brains were dissected. Frozen brains were cut into either 14 μm or 35 µm thick coronal sections. Following H&E staining (Histolab, Gothenburg, Sweden) or Toluidine blue staining (Sigmma-Aldrich), images of the sections were acquired using a light microscope (Zeiss Stemi 2000-C; Zeiss Gmbh, Göttingen, Germany or Leica DM6000B, Leica Microsystems). The injury-induced loss of brain tissue was measured in H&E stained section in each hemisphere with the SectionToVolume software (Hanell et al. 2012). The lesion area (mm^2^) was calculated by outlining missing cortical tissue for each section taken at 0.5 mm intervals, and lesion volume (mm^3^) determined by multiplying the sum of the contused areas obtained from each section by the distance between Sects. (0.5 mm). The tissue loss in the injured hemisphere after the left (mean ± S.E.M.: 13.55 ± 3.75 mm^3^; *n* = 5) and right (13.03 ± 4.14 mm^3^; *n* = 5) CCI was virtually identical. Almost no tissue was lost in the contralateral hemisphere after the left (0.02 ± 0.00 mm^3^; *n* = 5) and right (0.01 ± 0.00 mm^3^; *n* = 5) CCI, and in both hemispheres after the left (0.01 ± 0.00 mm^3^; *n* = 3) and right (0.01 ± 0.00 mm^3^; *n* = 3) sham injury. The architecture of the brain structures around the lesion site was visualized in [Fig. 1a–c (also see Fig. 1 in (Watanabe et al. 2020)]. The extent of the CCI injury ranged 2–4 mm rostrocaudally, 2–4 mm mediolaterally, and 1.5–2 mm in depth. No cortical injury was observed in sham-injured rats.

#### Unilateral ablation brain injury (UBI)

The scalp was incised and opened, a section of cranium located 0.5–4.0 mm posterior to the bregma and 1.8–3.8 lateral to the midline on the left or right side was removed, and the part of the cerebral cortex located below the opening that includes the hindlimb representation area of the sensorimotor cortex was aspirated with a metallic pipette (tip diameter 0.5 mm) (for details, see (Zhang et al. 2020)). For sham injury, animals underwent the same procedures, but the cortex was not ablated.

Localization and size of cortical lesions, and their comparison between the left and right side UBI rats were described in our previous papers (Watanabe et al. 2020; Zhang et al. 2020; Watanabe et al. 2021) (Fig. 1a–d in (Zhang et al. 2020)). Briefly, the brains were cut into 50 µm think sections and stained with thionine. The lesion sites extended 3.2–5.0 mm rostrocaudally and 1.8–2.8 mm mediolaterally. The lesion was 1.0–1.5 mm in depth and did not affect the white matter below the cortex (Fig. 1a, b, d). The actual lesion size in some animals was slightly larger than intended (that was around 3.5 × 2 mm) due to tissue necrosis around the cavity border. The lesion volumes were not corrected for tissue shrinkage due to the fixation. The volumes were similar (*P* = 0.84, two tailed *t *test) between the left (mean ± S.E.M.: 6.13 ± 0.41 mm^3^; *n* = 9) and right (5.98 ± 0.64 mm^3^; *n* = 9) UBI rats.

### Analysis of the lateral body bending

**Fig. 2:**
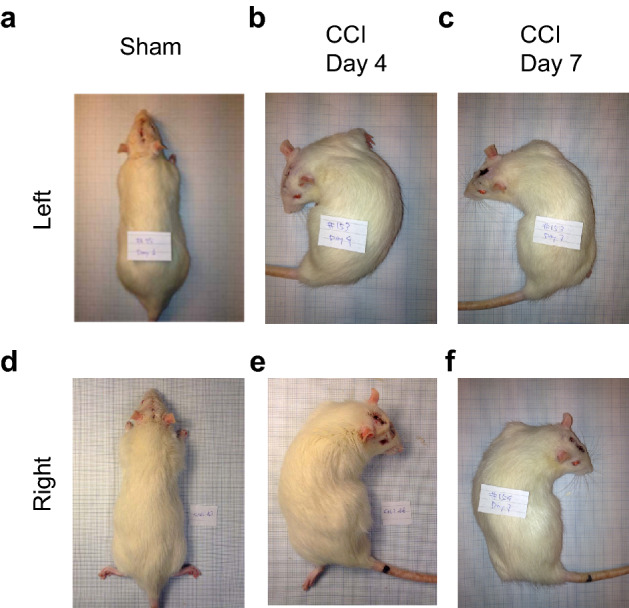
The left and right CCI-induced body bending revealed under ketamine anesthesia. Rats with sham injury (**a**, **d**) or CCI (**b**, **c**, **e**, and **f**) were analyzed on Day 4 (**a**, **b**, **d** and **e**) or Day 7 (**c**, **f**). CCI and sham surgery were performed either on the left (**a**–**c**) or (**d**–**f**) right side. Rats were categorized as symmetric (not bent), or laterally bent from the neck to the tail to the left or to the right in the coronal plane. The number of rats is shown in Table 1. All rats in each group displayed virtually the same either symmetric or asymmetric body posture at each of the 10, 20, and 30 min time points after ketamine administration

After administration of ketamine (60 mg/kg; intraperitoneally, i.p.), the rats were placed on the 1-mm grid paper in the prone position, the body was straightened in the rostro-caudal direction and then set free. The body posture was photographically recorded 10, 20 and 30 min after the injection and assessed as symmetric (not bent), or laterally bent to the left or to the right in the coronal plane. After the third measurement, additional dose of ketamine (10–20 mg/kg) supplemented with xylazine, an α_2_-adrenergic receptor agonist (5 mg/kg; i.p.), was injected and 2–5 min later the body posture was photographically recorded. In a subset of rats, the first injection of ketamine was supplemented with xylazine (5 mg/kg; i.p.). Typical left and right bent rats are shown in Fig. 2. The body bending was analyzed on Day 3 or 4, and Day 7 after CCI or sham injury.

### Spinal cord transection

The transection procedure was previously described (Watanabe et al. 2020, 2021; Zhang et al. 2020). Briefly, animals were anesthetized with sodium pentobarbital, and after measurement of postural asymmetry a laminectomy at the T2–T3 level was carried out. The spinal cord was doubly ligated and then completely transected between the ligatures. The completeness of the transection was confirmed by (i) inspecting the cord under microscope during the operation to ensure that no spared fibers bridged the transection site and that the rostral and caudal stumps of the spinal cord are completely retracted; (ii) placing a piece of Spongostan (Medispon^®^ (MDD, Toruń, Poland) between the rostral and caudal stumps of the spinal cord; and (iii) examining the spinal cord after termination of the experiment. In a subset of rats, the 3–4-mm spinal cord segment was dissected and removed; the following microscopic and functional analyses demonstrated that the transection was complete, and that the brain injury-induced HL-PA was of the same size and direction as before the spinal transection (for details, see (Lukoyanov et al. 2020)).

### Analysis of HL-PA

The magnitude of HL-PA and the side of the flexed limb were assessed as described previously (Bakalkin and Kobylyansky 1989; Watanabe et al. 2020; Zhang et al. 2020). Briefly, the measurements were performed under pentobarbital anesthesia (60 mg/kg, i.p.). The level of anesthesia was characterized by a barely perceptible corneal reflex and a lack of overall muscle tone. The rat was placed in the prone position on the 1-mm grid paper, and the hindlimbs were straightened in the hip and knee joints by gently pulling them backwards for 5–10 mm to reach the same level. Then, the limbs were set free and the magnitude of HL-PA was measured in millimeters as the length of the projection of the line connecting symmetric hindlimb distal points (digits 2–4) on the longitudinal axis of the rat (see Fig. 1e, f in (Watanabe et al. 2020)). The procedure was repeated six times in immediate succession. The limb displaying shorter projection was considered as flexed. The HL-PA magnitude in millimeters and the flexion side were used in statistical analysis. HL-PA was analyzed before and 60 min after spinal cord transection on Day 3 or Day 4 after CCI, UBI, or sham injury.

### Statistical analysis

Processing and statistical analysis of HL-PA was performed after completion of the experiments by the statistician (D.S.), who was not involved in execution of experiments. Therefore, the results of intermediate statistical analyses could not affect acquisition of experimental data. The HL-PA magnitude in millimeters and the flexion side were analyzed using Bayesian regression models via full Bayesian framework by calling *Stan* 2.21 (Carpenter et al. 2017) from *R* 3.6.3 R Core Team using the *brms* 2.12 (Burkner 2017) interface. Predictors and outcomes were centered and scaled. To reduce the influence of outliers, models used Student’s *t* response distribution family with identity link function. Models had no intercepts with indexing approach to predictors (McElreath 2019). According to Stan recommendations (Stan Development Team 2019) weakly informative priors were used for group-level effects, residual SD and group-level SD. *P* values, adjusted using the multivariate *t* distribution with the same covariance structure as the estimates, were produced by frequentist summary in *emmeans* 1.4.6 (Searle et al. 1980). Medians of the posterior distribution and 95% highest posterior density continuous intervals were plotted. The contrast between groups was defined as significant if both 95% highest posterior density continuous intervals did not include zero and adjusted *P* value was ≤ 0.05.

## Results

### CCI-induced body bending

The effects of the unilateral CCI were analyzed in animals under ketamine, ketamine and xylazine, pentobarbital or isoflurane anesthesia. As reported previously (Hetzler and Wautlet 1985; Winters et al. 1986) administration of an anesthetic doses of ketamine produced a cataleptic immobility. Unexpectedly, the CCI rats injected with ketamine displayed strong consistent bending of the body from neck to tail to the left or to the right in the coronal plane (Fig. 2; Table 1). Intact rats and animals with the sham injury did not develop such asymmetric responses. The rats with the left CCI were bent to the left with the concave and convex on the left and right side, respectively. The effects of the right CCI were mirror symmetric to those of the left brain trauma. The bending response was evident at all three time points 10, 20 and 30 min after ketamine administration, and was virtually the same on Day 4 and Day 7 after the CCI. All animals in each group demonstrated bending to virtually the same degree and direction (Fig. 2; Table 1). The body of the CCI rats was curved possibly due to muscle contraction on the concave side complemented by muscle stretching on the convex side that laterally flexed the spine. Administration of xylazine, an α_2_-adrenergic receptor agonist with analgesic and muscle relaxant properties, in combination with ketamine before the analysis, or after the third measurement of rats under ketamine anesthesia prevented and resolved the bending of the body, respectively. The bending was not observed in the CCI rats under pentobarbital or isoflurane anesthesia.

**Table 1:** The CCI effects on the body posture in the coronal plane analyzed under ketamine anesthesia

Surgery and treatment	Surgery side	The number of rats	Symmetric rats	Left bent rats	Right bent rats
Intact rats		6	6	0	0
Sham injury	Left	3	3	0	0
CCI	Left	6	0	6	0
CCI + xylazine^a^	Left	3	3	0	0
Sham injury	Right	4	4	0	0
CCI	Right	8	0	0	8

The posture was assessed as symmetric (not bent), or laterally bent to the left or to the right in the coronal plane. All rats in a group were either symmetric or laterally bent at the 10, 20, and 30 min time point after ketamine administration. Xylazine co-administered with ketamine before the analysis (^a^), or injected 30 min after ketamine resolved the CCI-induced asymmetry in all treated animals. The same bending pattern was evident on Day 4 and Day 7 after the CCI in each rat

^a^Xylazine was co-administered with ketamine before the analysis

### The left CCI-induced HL-PA: ipsilesional flexion and spinal fixation

**Fig. 3:**
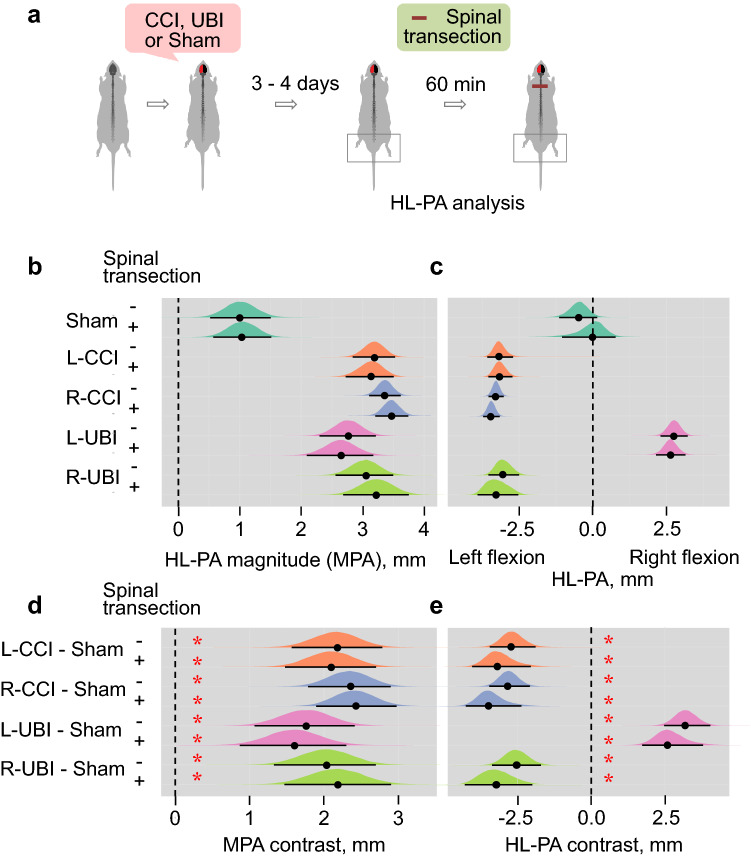
The HL-PA induced by the left- (L-) or right- (R-) side CCI, and by the left- or right side UBI. **a** Experimental design. HL-PA was analyzed before [shown as (−)] and 60 min after [shown as (+)] spinal cord transection at the T2-3 level on the Day 3 or Day 4 after CCI, UBI or sham injury; the effects of the left CCI are shown. **b**, **c** The magnitude of HL-PA (MPA) and HL-PA size in millimeters (mm), respectively. In **c**, negative and positive HL-PA values are assigned to rats with the left and right hindlimb flexion, respectively. **d**, **e** Differences (contrasts) between the CCI and sham injury groups, and between the UBI and sham injury groups in the asymmetry magnitude and its size, respectively. The number of animals is given in Table 2. The MPA, HL-PA, and the contrasts are plotted as median (black circles), 95% highest posterior density continuous intervals (black lines) and posterior density from Bayesian regression. *Significant differences (contrasts) between the groups: 95% highest posterior density continuous intervals did not include zero value, and adjusted *P* values identified by Bayesian regression were ≤ 0.05. Adjusted *P* values are shown in Online Resource 1 and Online Resource 2

**Fig. 4:**
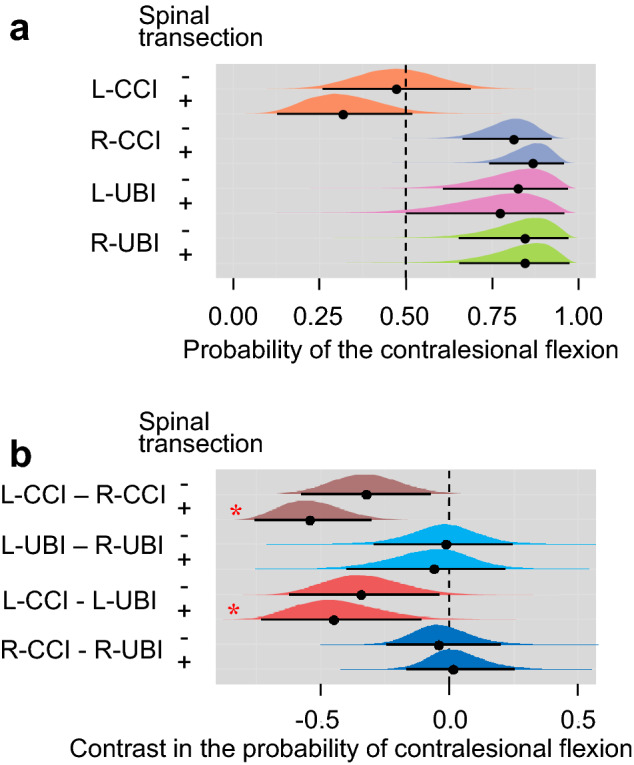
The probability of contralesional flexion in the left- (L-) and right- (R-) side CCI and the left- and right-side UBI groups. **a** The probability is plotted as median (black circles), 95% highest posterior density continuous intervals (black lines) and posterior density from Bayesian regression. *Significant differences (contrasts) between the groups: 95% highest posterior density continuous intervals did not include zero value, and adjusted *P* values identified by Bayesian regression were ≤ 0.05. Adjusted *P* values are shown in Online Resource 3. For details, see legend to Fig. 3

We previously demonstrated that the right CCI along with the left and right UBI produced HL-PA with flexion of the contralateral hindlimb that was evident under pentobarbital or isoflurane anesthesia (Zhang et al. 2020; Watanabe et al. 2021). In the course of HL-PA analysis, we examined whether the left CCI also induced HL-PA, whether contralesional hindlimb was flexed, and whether HL-PA persisted after spinal cord transection. The HL-PA was analyzed under pentobarbital anesthesia before and 60 min after complete spinal cord transection at the T2-T3 level on Day 3 or Day 4 after the left CCI or sham injury (Fig. 3 and 4; Tables 2 and Online Resources 1–3). The right CCI rats, and the left and right UBI animals were analyzed for comparison. Differences between animal groups for the magnitude of postural asymmetry (MPA) and HL-PA size were assessed by Bayesian statistics. The left CCI rats developed HL-PA that was evident both before and after spinal cord transection, while HP-PA was not observed in sham-injured rats. HL-PA with similar MPA values was developed after the right CCI, and left and right UBI as reported previously (Zhang et al. 2020; Watanabe et al. 2021). The MPA in each of four groups that received brain injury was different from MPA in sham injury rats (Fig. 3b, d; Tables 2 and Online Resource 1). Consistent with the previous reports, the left hindlimb was flexed in the right CCI and right UBI rats, while the left UBI produced right flexion (Fig. 3c) (Zhang et al. 2020; Watanabe et al. 2021). Unexpectedly, and in contrast with these reports, rats with the left CCI developed left limb flexion (Fig. 3c, e; Online Resource 2). Effects of brain injuries in all four groups were different from those of sham injury with high significance. The HL-PA with virtually the same magnitude and direction were evident in rats analyzed before the spinal cord transection.

**Table 2:** The number of rats analyzed for development of HL-PA

Groups	Spinal transection	
	Before	After
Sham injury	10	10
L-CCI	15	15
R-CCI	27	27
L-UBI	8	5
R-UBI	10	10

The probability to develop contralesional flexion was high in the right CCI and left and right UBI groups, while low in the left CCI rats (Fig. 4a). The left CCI group differed in this probability from the right CCI group and left UBI group after but not before spinal cord transection (Fig. 4b; Online Resource 3).

## Discussion

The main finding of the present study was that the left and right CCI induced HL-PA with the left hindlimb flexion that was, respectively, ipsi- and contralateral to the injured hemisphere. Ipsilesional effects of a unilateral brain lesion, traumatic brain injury, and stroke were documented in animal experiments and clinical studies (Gonzalez et al. 2004; Varghese and Winstein 2019; Maenza et al. 2020). In stroke survivors, functional deficits of ipsilesional limb depended on two factors, namely the side of damage and severity of contralesional impairments (Varghese and Winstein 2019; Maenza et al. 2020). The most severe ipsilesional deficits were expressed in patients with left side stroke and severe contralesional impairments. Findings of this study corroborate these clinical observations by demonstration that there are components or signs of sensorimotor and postural deficits that are induced ipsilaterally by the left side brain lesion while the right side injury produces contralesional effects.

### Putative neural mechanism of HL-PA and body bending

**Fig. 5:**
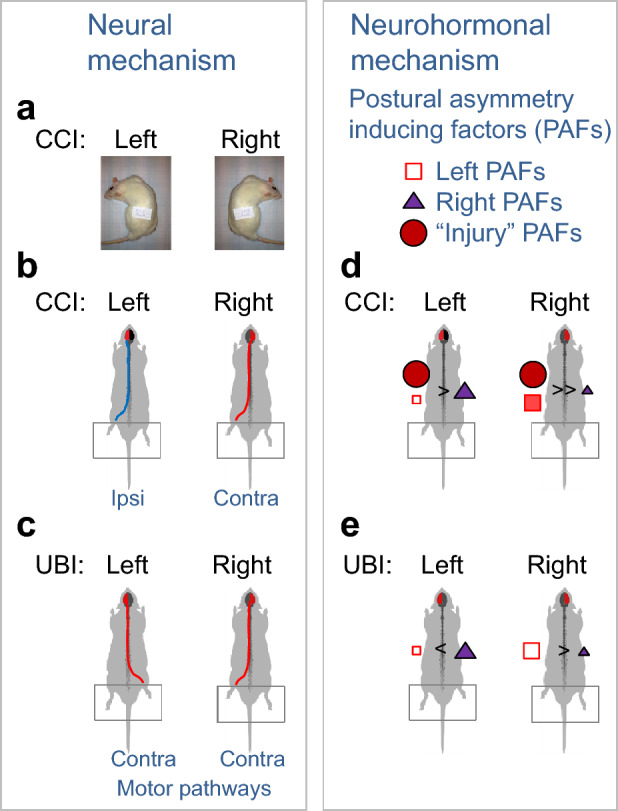
Hypothetical neural and neurohormonal mechanisms of contralesional and ipsilesional responses induced by the left and right CCI, and the left and right UBI. **a–c** Descending motor tracts may mediate effects of the brain injury on body bending and HL-PA. **a** Lateral body bending directed to the ipsilesional side after the left- and right-side CCI, and **c** formation of the contralesional hindlimb flexion after the left- and right-side UBI may be mediated by decussating neural tracts that are mirror-symmetrical. **b** Ipsilateral and decussating contralateral motor pathways may be engaged in formation of HL-PA with left hindlimb flexion after the left- and right-side CCI, respectively; the left CCI in contrast to the left UBI and right CCI may activate the ipsilateral motor pathway. This pathway may control hindlimb responses but not body bending, and may be anatomically asymmetric because the right CCI instead produces the contralesional hindlimb flexion. **d**, **e** Two groups of neurohormones and neuropeptides may produce HL-PA with flexion of the left or right hindlimb, respectively (Bakalkin et al. 1989; Watanabe et al. 2020, 2021). Activity of these left and right postural asymmetry inducing factors (PAFs) may be balanced in the symmetric brain, while CCI and UBI may impair the balance and shift an equilibrium in PAFs activity to favor the factors producing either the contralesional or ipsilesional hindlimb response. Some of the PAFs, for example, endogenous opioid peptides dynorphins and Met-enkephalin are involved in tissue injury, pain and stress, and overproduced after brain trauma and spinal cord injury (Hauser et al. 2005; Hussain et al. 2012; Maximyuk et al. 2015; Smeets et al. 2015). The overproduction or excessive release of these injury-associated PAFs may be similar after the left and right side CCI. Similarly with synthetic dynorphins and Met-enkephalin that induce hindlimb flexion on the left side in animals with intact brain, the endogenous injury-associated PAFs may induce a unilateral response, namely the left hindlimb flexion (Bakalkin et al. 1989; Watanabe et al. 2020). In comparison with the CCI, the UBI does not produce a severe brain damage and, therefore, may not activate the injury-associated PAFs, while the side-specific PAFs may be generated and enable formation of the contralesional hindlimb flexion (Bakalkin et al. 1989; Watanabe et al. 2020)

Neural mechanism may underlie the left side response to the left and right side CCI and could be mediated by the ipsi- and contralateral descending pathways, respectively (Fig. 5). Different effects of the left and right CCI on corticospinal projecting neurons and descending pathways that signal to the ipsilateral spinal cord suggest that their anatomical organization differs between the left and right hemispheres. This neural asymmetry may be supported by human, rodent, and insect findings that demonstrated general lateralization of the motor system (Robinson 1979; Sainburg 2014; Knebel et al. 2018; Stancher et al. 2018; Zhang et al. 2020). At the same time, development of the contralesional hindlimb flexion induced by ablation injury to the left or right hemispheres [Fig. 5c and (Lukoyanov et al. 2020; Watanabe et al. 2020, 2021; Zhang et al. 2020)] suggests that the ipsilateral descending motor pathways are less affected by the UBI *vs*. the CCI in the left hemisphere.

In contrast to the left side hindlimb response developed under pentobarbital or isoflurane anesthesia, the left and right CCI rats displayed the mirror-symmetrical body bending patterns under ketamine anesthesia, suggesting that the injury effects were mediated through mirror symmetric neural pathways. To our knowledge the brain injury-induced postural effects that were not seen in unanesthetized animals but uncovered by administration of ketamine, have not yet been reported. Bulbospinal tracts specifically reticulospinal and vestibulospinal tracts innervate the ipsilateral axial and proximal limb muscles controlling posture and balance (Purves et al. 2012). Both tracts receive projections from sensorimotor cortex (McCall et al. 2017; Cregg et al. 2020), and may be affected by CCI and therefore their activity may be reduced on the contralesional side.

Ketamine, a non-competitive *N*-methyl-d-aspartate receptor antagonist, may interfere with synaptic transmission in these tracts that use glutamate as a neurotransmitter. The contra- and ipsilateral tracts may be differentially affected by the CCI and develop different sensitivity to ketamine. Their different inhibition by ketamine may result in stronger contraction of ipsi- *vs*. contralesional axial muscles leading to body bending. Alternatively, stimulation of the *N*-methyl-d-aspartate ion channel by endogenous glutamate may have a compensatory function and maintain a balance in activity of the affected and unaffected counterparts of mirror-symmetrical neural circuits in unanesthetized animals after a unilateral brain trauma. This could counteract the lateral deformation of the body, while inhibition of this mechanism by ketamine would allow the body bending in rats with CCI. Overall, the left and right CCI produced the mirror-symmetrical effects on the body bending, while the left side flexion was revealed in the HL-PA model and was apparently mediated by different neural circuits.

The asymmetric tonic neck reflex (ATNR) discovered by Magnus and de Kleijn is a primitive reflex that is present early in life and may reemerge in adults after brain injury (Shevell 2009). It was described as a “fencing” posture where rotation of the head results in an asymmetric muscle contraction pattern with extension of the limbs ipsilateral to the direction of head rotation, and flexion of the limbs contralateral to the direction of head rotation. In recent studies, evidence was provided for the re-emergence of ATNR after stroke and traumatic brain injury, and for its reliance on ipsilateral reticulospinal pathways as the mechanism (Ellis et al. 2012; McPherson et al. 2018). At the same time, a unilateral microstimulation of the reticular formation produced head movements that were directed ipsilaterally to both the side of stimulation and the side of activation of the upper limb flexors (Drew and Rossignol 1990; Quessy and Freedman 2004). In this experimental setting, the direction of head rotation was opposite to the ATNR. The effects induced by ketamine administration to the CCI rats resembled the postural pattern produced by microsimulation of the reticular formation in pairing of the head rotation to the ipsilesional side with bending of the body from neck to tail with the concave on the same side. This postural pattern may be induced through asymmetric effects of ketamine on the contra- and ipsilesional reticulospinal pathways differently affected by the CCI. The link of the ketamine effects with the ATNR requires further analysis. Formation of HL-PA was not apparently related to the ATNR. First, because the head was fixed in the symmetric position in these experiments, and second, because HL-PA persisted after complete spinal cord transection. Nonetheless, it would be interesting to examine whether the head rotation to or from the affected limb would produce any changes in the HL-PA.

### Side-specific neuroendocrine mechanism of HL-PA formation

The CCI and UBI differ in their pathophysiological mechanisms. CCI produces substantial and progressive injury to the white matter and underlying structures (Hall et al. 2005, 2008). In contrast, the lesion caused by the UBI is confined to the cortex. In the CCI, brain damage is caused by mechanical stress-induced axonal injury and vascular dysfunction followed by dispersed neuronal degeneration and death, neuroinflammation, and excitotoxicity (Hall et al. 2005, 2008; Ng and Lee 2019; Lacalle-Aurioles et al. 2020). These processes are mediated by a variety of endogenous regulatory molecules including neuropeptides, cytokines, and neurotrophic factors. These molecules may also shape functional connectivity within neural circuits and modify their functions (Bargmann 2012; Nusbaum et al. 2017). These injury-associated molecules may be activated by both the left or right side brain injury, while in intact brain some of them may be involved in lateralized processes and even produce side-specific effects (Deliagina et al. 2000; Hussain et al. 2012; Marlin et al. 2015; Nation et al. 2018; Phelps et al. 2019; Watanabe et al. 2020, 2021). For example, the opioid peptides dynorphins are pathogenic when they are overexpressed in response to chronic pain, and brain, and spinal cord injury (Caudle and Mannes 2000; Hauser et al. 2005; Hussain et al. 2012; Maximyuk et al. 2015; Smeets et al. 2015). Upregulated dynorphins may also induce asymmetric responses including development of HL-PA (Bakalkin and Kobylyansky 1989; Watanabe et al. 2020, 2021). We previously demonstrated that administration of opioid antagonists to rats with a unilateral brain lesion inhibited HL-PA formation, while opioid peptides and synthetic opioids mimicked effects of brain injury by inducing HL-PA in rats with intact brain (Chazov et al. 1981; Bakalkin et al. 1986; Bakalkin and Kobylyansky 1989; Watanabe et al. 2020, 2021). The right UBI-induced left flexion may be mediated by dynorphins and Met-enkephalin, while the left UBI-induced HL-PA with right flexion may be mediated by Leu-enkephalin (Chazov et al. 1981; Bakalkin et al. 1986; Bakalkin and Kobylyansky 1989; Watanabe et al. 2020, 2021).

In the context of the HL-PA neurohormonal mechanism (Fig. 5d, e), the left side response to the left and right side CCI may be developed due to overproduction or excessive release of pathogenic dynorphins, Met-enkephalin or other neurohormones with the left side actions (Fig. 5d). The “left side” neurohormones may prevail over the “right side” regulatory factors after both the left and right CCI but not after UBI. The UBI mechanistically differs from the CCI and may not activate these neurohormonal systems at least to the same extent. Neural pathways or neurohormonal systems that are differently activated by the left and right UBI may dominate in signaling from the injured brain to the lumbar neural circuits controlling the right and left hindlimb muscles, respectively (Fig. 5e).

### Limitations

The limitations of this study were first that the CCI effects were examined in anesthetized animals. Still, the analysis was valid; anesthesia with isoflurane and pentobarbital did not interfere with HL-PA magnitude and flexion side as it was evident from analyses of anesthetized animals and unanesthetized decerebrate rats in our previous studies (Lukoyanov et al. 2020; Zhang et al. 2020). Second, the study focused on the HL-PA and body bending while did not evaluate pathophysiological and neuroendocrine mechanisms of postural deficits. However, the HL-PA has been validated in our previous studies by analysis of the hindlimb resistance to stretch, hindlimb withdrawal reflexes evoked by electrical stimulation and recorded with EMG technique, and hindlimb motor deficits in locomotor tasks in rats with UBI (Lukoyanov et al. 2020; Zhang et al. 2020; Watanabe et al. 2021). The contra- and ipsilesional hindlimbs displayed different musculo-articular resistance to stretch and different reflex activation patterns after the brain injury. The direction of the UBI-induced effects perfectly correlated among the HL-PA, the stretching resistance and EMG asymmetries, and asymmetric hindlimb motor impairments in locomotor tasks. Also, the HL-PA magnitude and the contra-ipsilesional differences in the stretching resistance strongly correlated. We did not identify the afferent and efferent neural circuits involved, and neural and neurohormonal pathways that signal from the injured brain to spinal cord. On the other hand we demonstrated that the HL-PA modelled several pathophysiological features of the upper motor neuron syndrome induced by traumatic brain injury or stroke primarily their asymmetric patterns (Lukoyanov et al. 2020; Zhang et al. 2020; Watanabe et al. 2021). Furthermore, we showed the HL-PA was blocked by a muscle relaxant while the asymmetry was resistant to bilateral lumbar deafferentation suggesting the mechanism dependent on the efferent drive but not on afferent input (Zhang et al. 2020). In this instance, HL-PA is arguably similar to “spastic dystonia”, a tonic muscle overactivity that contributes to “hemiplegic posture” in patients with stroke or cerebral palsy (Gracies 2005; Sheean and McGuire 2009; Lorentzen et al. 2018).

## Conclusions

This study describes an unusual left-side hindlimb response to a unilateral traumatic brain injury that does not depend on the side of the lesion. The mirror symmetry was disrupted in the development of HL-PA that was induced by traumatic injury of either the left or right hemisphere. At the same time, mirror-symmetrical postural responses were developed after the localized ablation cortical injury in the HL-PA model, and after the CCI in body bending. Lateralized neural and side-specific neurohormonal mechanisms may signal from an injured hemisphere to spinal neural circuits and account for the disrupted mirror symmetry in effects of the left and right side CCI. These mechanisms may have a role in development of the ipsilesional *vs*. contralesional sensorimotor and postural deficits secondary to traumatic brain injury and stroke.

## Supplementary Information

Below is the link to the electronic supplementary material.

Supplementary file1 (DOCX 47 KB)

## Data Availability

Data supporting the findings of this study are available within the article, its Online Resources, or upon request.

## Abbreviations

ATNR: Asymmetric tonic neck reflex
CCI: Controlled cortical impact
HL-PA: Hindlimb postural asymmetry
MPA: Magnitude of postural asymmetry
PAFs: Postural asymmetry inducing factors
