# Supplementary material for: Unilateral traumatic brain injury of the left and right hemisphere produces the left hindlimb response in rats
Source: Exp Brain Res. 2021 May 22;239(7):2221–32. doi: 10.1007/s00221-021-06118-4 (PMC8282563; doi:10.1007/s00221-021-06118-4)
Supplement: Supplementary file 1 — Supplementary file1 (DOCX 47 KB) [file 221_2021_6118_MOESM1_ESM.docx]

**Unilateral traumatic brain injury of the left and right hemisphere produces the left hindlimb response in rats**

***Experimental Brain Research***

**Georgy Bakalkin, Olga Nosova, Daniil Sarkisyan, Mathias Hallberg, Mengliang Zhang, Jens Schouenborg, Niklas Marklund and Hiroyuki Watanabe**

Correspondence to:

Georgy Bakalkin

Department of Pharmaceutical Biosciences

Uppsala University

Husargatan 3, Box 591

751 24 Uppsala, Sweden

[Georgy.Bakalkin@farmbio.uu.se](mailto:Georgy.Bakalkin@farmbio.uu.se)

**Online Resource 1**

Analysis of the HL-PA magnitude using Bayesian statistics. Graphical data are shown on Fig. 3b,d. Estimate (median), lower and upper values of 95% posterior density continuous intervals (HPD), and adjusted P-value are shown. Sham, sham injury group.

| **Spinal transection** | **Contrast** | **Estimate (median)** | **Lower HPD** | **Upper HPD** | **P value** |
| --- | --- | --- | --- | --- | --- |
| Before | L-CCI - Sham | 2.174 | 1.566 | 2.783 | 1.70e-11 |
| Before | R-CCI - Sham | 2.351 | 1.785 | 2.896 | 1.11e-15 |
| Before | L-UBI - Sham | 1.753 | 1.066 | 2.412 | 8.85e-06 |
| Before | R-UBI - Sham | 2.028 | 1.326 | 2.696 | 1.11e-07 |
| After | L-CCI - Sham | 2.090 | 1.475 | 2.698 | 1.32e-10 |
| After | R-CCI - Sham | 2.422 | 1.894 | 2.975 | <1.00e-10 |
| After | L-UBI - Sham | 1.597 | 0.868 | 2.299 | 1.02e-04 |
| After | R-UBI - Sham | 2.179 | 1.468 | 2.901 | 1.54e-08 |

**Online Resource 2**

Analysis of the HL-PA size using Bayesian statistics. Graphical data are shown on Fig. 3c,e. Estimate (median), lower and upper values of 95% posterior density continuous intervals (HPD), and adjusted P-value are shown. Sham, sham injury group.

| **Spinal transection** | **Contrast** | **Estimate (median)** | **Lower HPD** | **Upper HPD** | **P value** |
| --- | --- | --- | --- | --- | --- |
| Before | L-CCI - Sham | -2.690 | -3.448 | -1.893 | 2.65e-10 |
| Before | R-CCI - Sham | -2.808 | -3.469 | -2.088 | 6.22e-15 |
| Before | L-UBI - Sham | 3.225 | 2.476 | 4.049 | 3.00e-15 |
| Before | R-UBI - Sham | -2.561 | -3.371 | -1.712 | 1.02e-08 |
| After | L-CCI - Sham | -3.157 | -4.049 | -2.055 | 3.74e-09 |
| After | R-CCI - Sham | -3.457 | -4.266 | -2.372 | 5.70e-12 |
| After | L-UBI - Sham | 2.625 | 1.728 | 3.800 | 3.96e-06 |
| After | R-UBI - Sham | -3.252 | -4.298 | -2.007 | 7.39e-07 |

**Online Resource 3**

Contrasts in the probability to develop contralesional flexion between the left- (L) and right (R) CCI groups, left- and right UBI groups, and CCI and UBI groups assessed by Bayesian statistics. Graphical data are shown on Fig. 4. Estimate (median), lower and upper values of 95% posterior density continuous intervals (HPD), and adjusted P-value are shown.

| **Spinal transec tion** | **Contrast** | **Estimate (median)** | **Lower HPD** | **Upper HPD** | **P value** |
| --- | --- | --- | --- | --- | --- |
| Before | L-CCI – L-UBI | -0.339 | -0.622 | -0.039 | 1.40e-01 |
| Before | R-CCI – R-UBI | -0.036 | -0.244 | 0.197 | 1.00e+00 |
| After | L-CCI – L-UBI | -0.445 | -0.732 | -0.109 | 3.93e-02 |
| After | R-CCI – R-UBI | 0.019 | -0.167 | 0.252 | 1.00e+00 |
| Before | L-CCI – R-CCI | -0.327 | -0.577 | -0.072 | 8.95e-02 |
| Before | L-UBI – R-UBI | -0.020 | -0.293 | 0.245 | 1.00e+00 |
| After | L-CCI – R-CCI | -0.544 | -0.757 | -0.303 | 3.27e-05 |
| After | L-UBI – R-UBI | -0.067 | -0.399 | 0.215 | 9.98e-01 |
